# The neurovascular unit in leukodystrophies: towards solving the puzzle

**DOI:** 10.1186/s12987-022-00316-0

**Published:** 2022-02-28

**Authors:** Parand Zarekiani, Henrique Nogueira Pinto, Elly M. Hol, Marianna Bugiani, Helga E. de Vries

**Affiliations:** 1 Department of Pathology, Amsterdam Neuroscience, Amsterdam UMC, Vrije Universiteit Amsterdam, de Boelelaan 1117, Amsterdam, The Netherlands; 2 Amsterdam Leukodystrophy Center, Amsterdam UMC, Amsterdam, The Netherlands; 3 Department of Molecular Cell Biology and Immunology, Amsterdam Neuroscience, Amsterdam UMC, Vrije Universiteit Amsterdam, De Boelelaan 1117, Amsterdam, The Netherlands; 4 Department of Translational Neuroscience, University Medical Center Utrecht Brain Center, Utrecht University, Utrecht, The Netherlands

**Keywords:** Neurovascular unit, Blood–brain barrier, Leukodystrophies, In vitro models, Induced pluripotent stem cells, Endothelium, Astrocyte, Pericyte, Microglia

## Abstract

The neurovascular unit (NVU) is a highly organized multicellular system localized in the brain, formed by neuronal, glial (astrocytes, oligodendrocytes, and microglia) and vascular (endothelial cells and pericytes) cells. The blood–brain barrier, a complex and dynamic endothelial cell barrier in the brain microvasculature that separates the blood from the brain parenchyma, is a component of the NVU. In a variety of neurological disorders, including Alzheimer’s disease, multiple sclerosis, and stroke, dysfunctions of the NVU occurs. There is, however, a lack of knowledge regarding the NVU function in leukodystrophies, which are rare monogenic disorders that primarily affect the white matter. Since leukodystrophies are rare diseases, human brain tissue availability is scarce and representative animal models that significantly recapitulate the disease are difficult to develop. The introduction of human induced pluripotent stem cells (hiPSC) now makes it possible to surpass these limitations while maintaining the ability to work in a biologically relevant human context and safeguarding the genetic background of the patient. This review aims to provide further insights into the NVU functioning in leukodystrophies, with a special focus on iPSC-derived models that can be used to dissect neurovascular pathophysiology in these diseases.

## Background

The neurovascular unit (NVU) is a complex dynamic structure present in the microvasculature of the central nervous system (CNS). It is comprised of brain endothelial cells (BECs), pericytes, astrocytes, microglia, and neurons. Its proper function is key for the maintenance of brain homeostasis by separating the blood from the CNS [1]. Also, the NVU is responsible for neurovascular coupling (NVC), which is the mechanism that adjusts local blood supply to neuronal demand through changes in vascular intraluminal diameter [1].

At the capillaries, the first line of defence is a layer of specialized polarized BECs, which are sealed together by protein complexes that form adherens junctions (AJs) comprised of VE-Cadherin, and tight junctions (TJs), formed by claudins, occludin and other cytoplasmic plaque proteins. This endothelial structure is referred to as the blood–brain barrier (BBB). BECs lack fenestrae and have low rates of transcytosis, thereby disabling transcellular and paracellular routing of molecules into the CNS. Metabolites, nutrients, and (large) essential molecules are actively transported across the BECs into the CNS by specific polarized transporters. In turn, toxins, xenobiotics, and waste products are actively removed from the CNS by another class of polarized transporters, i.e. ATP binding cassette (ABC) transporters. Additionally, BECs physical barrier properties regulate infiltration of peripheral immune cells into the CNS to modulate the adaptive neuroinflammatory response [2–9].

The second cell type involved in the regulation of the NVU is the pericyte. These cells share the inner basement membrane with BECs and are directly connected to the endothelial cells via peg-socket junctions, which are composed of connexins and N-cadherin [10]. Via these peg-socket junctions, there is a direct exchange of ions, metabolites, and other small molecules. Another important function of pericytes is the regulation of the vessel diameter, which in its turn regulates the cerebral blood flow (CBF) [11, 12]. Subsequently, the CBF rate can influence the rate of exchange of molecules crossing the BBB.

The next cellular component of the NVU is the astrocyte. Within the CNS, astrocytes have multiple functions, including maintenance of ion-water homeostasis, support of myelination, regulation of glutamate transport and synthesis, enabling synaptic plasticity, control of immune reactions, and promotion of neurite outgrowth [13–16]. At the NVU, perivascular astrocytes are connected with their endfeet to the outer (glial) basement membrane. These endfeet are specialized and polarized structures containing orthogonal arrays of intramembranous particles (OAPs), which display clusters of the selective water channel aquaporin-4 (AQP4) and the ATP-sensitive potassium channel Kir 4.1. [17]. Under physiological conditions, AQP4 is mainly localized at astrocyte endfeet, regulating water uptake and clearance of the brain parenchyma [16]. Dystrophin-associated proteins, e.g. dystroglycans, and extracellular matrix (ECM) molecules, i.e. laminin and agrin, are crucial in anchoring AQP4 at the astrocytic endfeet [18–22]. Upon traumatic brain injury and/or oedema, astrocytes become reactive and redistribute AQP4 away from astrocytic endfeet [23]. These AQP4 water channels facilitate ion-water homeostasis at the NVU [2, 23–25]. Furthermore, astrocyte-derived soluble factors control the TJ and transporter expression in BECs and therefore regulate NVU function [24].

Additionally, the basement membrane is a highly organized structure within the NVU. It is comprised of ECM molecules as integrins, laminins, collagen and fibronectin. These are expressed and secreted by the various NVU cellular compartments dependent on the cues from the microenvironment [26]. Finally, microglia also participate in the NVU [27, 28]. In the developing CNS, microglia regulate the formation of CNS vasculature and the control the neuronal progenitor cell niche [29–31].

Not only is the NVU is a complex structure by itself, but it is also heterogeneous throughout brain regions. Each component of the NVU itself has a unique transcriptome, proteome and epigenetic profile depending on the developmental origin and brain region where it is situated [32–37]. Another important aspect regarding the heterogeneity throughout the human brain is that the capillary density is higher in grey matter (GM) compared to white matter (WM). This finding correlates with energy demand [38].

A large part of the human brain consists of WM and is responsible for establishing the neuroconnectivity that underlies the highly complex and unique behavioral capacities of humans [39, 40]. The WM comprises myelinated axons, diverse types of glial cells, and blood vessels. The WM is selectively affected in the class of leukodystrophies. Leukodystrophies are characterized by primarily affected WM regardless of the molecular processes involved and the disease course [41]. Before this new definition, leukodystrophies were seen as progressive WM disorders caused by a genetic defect, where myelin was the primary affected structure. The myelin defect observed was either a direct effect on myelin or indirect on oligodendrocytes, the myelin forming cells [42]. Later, magnetic resonance imaging (MRI) pattern recognition paved the way for stratifying patients and subsequent genetic explorations [43]. In the following decades, sequencing techniques also evolved, pathological data became more available and new disease models emerged. These scientific developments have given an enormous boost to the field of leukodystrophies. Usually, the clinical course of leukodystrophies is progressive, and often eventually fatal. So far only symptomatic treatments are available. Therefore, unravelling the underlying mechanisms of these diseases is a priority. As mentioned, the WM comprises different cell types that create a complex network of signalling in synergy, yet each leukodystrophy is caused by a different genetic deficit that results in a distinct WM pathology. Notably, the genetic deficits underlying these diseases are not restricted to myelin- or oligodendrocyte-specific genes. Recent next-generation sequencing studies combined with MRI pattern recognition have shown that a predominant dysfunction of cell types other than oligodendrocytes may drive the WM pathology in leukodystrophies. To distinguish the underlying mechanisms, it is essential to identify distinct pathological hallmarks and the specific cell types affected in this heterogeneous group of diseases. Therefore, a new classification system for leukodystrophies has recently been proposed based on cellular pathology and pathogenic mechanisms [44].

Strikingly, some cellular components that are primarily affected, such as astrocytes, are part of the NVU. The function of the NVU, however, has been overlooked in these diseases. The problem in the diagnosis of leukodystrophies is that MRI with contrast agents is not always common practice in the clinic. Additionally, leakage of contrast agents in MR imaging only highlights gross abnormalities of the BBB. Especially when looking at heterogeneous diseases, such as leukodystrophies, certain features, such as BBB leakage of smaller molecules/ions and structural changes in the brain vasculature, can be overlooked. We recent showed that there is NVU involvement in different leukodystrophies regardless of MRI contrast enhancement or the main cellular component involved in the disease [45]. Furthermore, the tools to study these rare diseases are limited and rely mostly on clinical studies and post-mortem brain tissue. Nowadays, alternative techniques are available with the emerging field of human induced pluripotent stem cells (hiPSCs). Using hiPSCs, complex human neural cell models can be built, such as organs-on-chips and organoids, which better recapitulate the in vivo biology compared to other disease models.

The first aim of this review is to outline the implications for the dysfunction of NVU components in leukodystrophies following the leukodystrophy classification system and how these could be important for disease mechanisms. The second aim is to address the current tools to study NVU mechanisms in leukodystrophies and outline future directions.

## Leukodystrophies

Leukodystrophies are classified into several categories depending on the main cellular mechanism of WM injury and other pathological mechanisms that contribute to the disease progression [44]. In this section, we describe the different leukodystrophy classes, and the key components driving the pathology, and we review the knowledge on how the NVU can contribute to disease. The common denominator in leukodystrophies is selectively affected WM, ranging from lack of myelin to complete WM atrophy. Clinically and pathologically, leukodystrophies are highly heterogeneous, therefore the main MRI characteristics, clinical phenotype, and pathological hallmarks are summarized in Table 1.

**Table 1:** Characteristics of leukodystrophies

Leukodystrophy category	Disease	Abbreviation	Gene involved	Main cell type involved	Clinical phenotype	MRI characteristics	Contrast enhancement		Macroscopic pathology	Microscopic pathology	NVU involvement	References
Hypomyelinating	Pelizaeus-Merzbacher disease	PMD	*PLP1*	Oligodendrocytes	Onset in first months of life of pendular nystagmus, developmental delay and hypotonia	Inconstant signal on T1 and mild signal increase in T2 weighted images of WM compared to GM; WM atrophy	No		Small brains; large lateral ventricles; cerebellar, brainstem, and spinal cord WM appears greyish and are shrunken	Lack of myelin; no mature oligodendrocytes; increased astrogliosis and microgliosis	Redistribution of AQP4 in frontal WM; neuroinflammation with activated microglia	[45, 47, 52, 194–198]
Demyelinating	Metachromatic leukodystrophy	MLD	*ARSA*	Oligodendrocytes	Age-related. Impaired motor skills, behavioural and concentration problems in late-infantile and juvenile forms; psychiatric disorders, including schizophrenia, and cognitive decline in the adult form	Hyperintensities on T2-weighted images initially limited to the corpus callosum and then progressing to the periventricular and subcortical WM. Characteristic pattern of radiating stripes ("tigroid" pattern) with normal signal intensity within the abnormal WM	Yes, only in cranial nerves		The demyelinated WM appears greyish. Cerebellum, brainstem and optic nerves are atrophic	Demyelination with accumulation of sulfatides in glial cells, neurons and macrophages. Negative correlation between increased demyelination and reactive gliosis in the CNS	Redistribution of AQP4 in frontal WM. Lipid-laden macrophages in perivascular space of WM can release inflammatory mediators	[45, 55–58, 199–202]
Myelin vacuolization	*LARS2-*related leukodystrophy	N/A	*LARS2*	Astrocytes	Deafness early in life and developmental delay	Diffusely abnormal cerebral WM and elevated brain lactate levels on magnetic resonance spectroscopy	Yes		Enlarged lateral ventricles and rarefaction to cystic degeneration of the periventricular WM. U-fibres are spared	Loss of myelin and reactive astrocytes at the vasculature	Redistribution of ZO-1 and AQP4. Expression of laminin in perivascular astrocytes	[41, 45]
Astrocytopathies	Alexander disease	AxD	*GFAP*	Astrocytes	Infantile form, with macrocephaly, seizures, early cognitive and motor dysfunctions. Later onset form with bulbar signs, cognitive dysfunction and dysautonomia	T2-signal changes predominant in the anterior brain regions and medulla oblongata	Yes, at the midbrain ventricular lining and the cerebral hemispheric periventricular rim		Severe WM degeneration in the cerebrum, cerebellum and brainstem	RFs in cell bodies and processes of astrocytes, enriched at the endfeet. Enlarged astrocytes, lymphocytic infiltrates. Activated microglia	RFs, low expression of α-dystroglycan and redistribution of AQP4. RFs can also mechanically disrupt vascular function	[45, 61, 64, 65, 70–73, 203]
Astrocytopathies	Aicardi-Goutières syndrome	AGS	*TREX1*, *RNASEH2A*, *RNASEH2B*, *RNASEH2C*, *SAMHD1*, *ADAR1* or *IFIH1/MDA*	Astrocytes	Early-onset progressive loss of motor functions and severe intellectual impairment. Non-neurological symptoms. Elevated lymphocyte and IFN-α levels in CNS	Calcification of the basal ganglia, WM abnormalities in the frontotemporal brain regions	No	Small brains, atrophic WM, calcifications		Co-localization of IFN-α and CXCL10 with GFAP. Infiltrating T-cells and calcification in brain vasculature. Thickened tunica media and adventitia in the arterioles and precapillary vessels. Microangiopathy in the cerebral WM, cerebellum and striatum	Thickened tunica media and adventitia. Redistribution of ZO-1 and AQP4	[74–76, 204–208]
Astrocytopathies	Megalencephalic leukoencephalopathy with subcortical cysts	MLC	*MLC1* or *GLIALCAM*	Astrocytes	Early-onset, progressive cerebellar ataxia, mild cognitive decline and epilepsy. Variable disease severity	Abnormal T2 diffuse signal of the cerebral WM, swelling of the abnormal WM and subcortical cysts. Increased water content in the WM	No		Large, swollen brain with small lateral ventricles, subcortical cysts in the fronto-temporal regions	Astrocytosis in molecular layer. Vacuolized and thin myelin. Vacuoles in astrocytic endfeet. Fibrillary astrogliosis and scarce microglia activation	MLC1 and GlialCAM regulate ion-water homeostasis at the endfeet and the anchoring of other ion and water channels. Possible MLC1 role in inflammatory signalling at the NVU	[82–90, 209–211]
Microgliopathies	Adult-onset leukodystrophy with spheroids and pigmented glia	ALSP	*CSF1R*	Microglia	Adult-onset, progressive personality changes, dementia and spasticity. Heterogeneous phenotype	T2-hyperintense signal changes in the periventricular, deep and subcortical cerebral WM. Cerebral white matter atrophy	No		WM atrophic and greyish	WM vacuolization and myelin loss. Swollen axons and axonal spheroids. Reactive gliosis, scarce microglia activation, lipid-laden macrophages and pigmented glia	Reactive gliosis and redistribution of AQP4 in frontal WM. IL-34-related mechanisms. Oxidative stress	[95, 97–99, 212, 213]
Leuko-vasculopathies	Cerebral autosomal dominant arteriopathy with subcortical infarcts and leukoencephalopathy	CADASIL	*NOTCH3*	VSMCs and pericytes	Adult-onset. Migraine aura, acute stroke syndromes, cognitive impairment, gait dysfunction, mood and behavioural changes	WM lesions and lacunar infarction. T2 hyperintensity deep WM, internal and external capsules, and temporal pole. Enlarged perivascular spaces. Cerebral microbleeds and brain atrophy	Yes		WM atrophy, U-fibres spared	Thickened vascular walls. Degeneration of VSMCs. Accumulation of fibrous ECM, narrowed blood vessels. Loss of myelin. Reactive astrogliosis	Granular osmiophilic material at the capillaries, microfilament bundles in BECs. AQP4 redistribution	[109, 114–118, 121, 214]
Leuko-vasculopathies	Cathepsin A-related arteriopathy with strokes and leukoencephalopathy	CARASAL	*CTSA*	Blood vessels	Adult-onset headaches or migraine, mild intellectual impairment and mild gait problems. Most patients develop hypertension, short ischemic attacks and strokes. Symptoms of brainstem dysfunction	Diffuse WM signal changes with infarcts and microbleeds in the basal nuclei, brainstem and cerebellum	No		Mild atrophy of the cerebral WM and small infarcts in subcortical and deep cerebral WM, basal nuclei, thalami, brainstem and cerebellum	Preserved axons, loss of myelin, astrogliosis and preserved oligodendrocytes. Loss of SMCs in the cerebral WM and basal nuclei. Upregulation of ET-1 in WM astrocytes	Reactive gliosis and redistribution of AQP4 in frontal WM. Prominent loss of SMA, thickening of basal lamina and occlusion of cerebral blood vessels	[123–125, 132]

Clinical and pathological features of the described leukodystrophies per category

Hypomyelinating leukodystrophies are characterized by an impaired developmental myelination in the CNS and possibly also the peripheral nervous system (PNS). Leukodystrophies in this category are both clinically and genetically heterogeneous, yet show similarities [46]. The prototypical hypomyelinating leukodystrophy is Pelizaeus-Merzbacher disease (PMD). PMD is an X-linked disorder caused by changes in *PLP1*, encoding proteolipid protein 1 (PLP1) and the alternative spliced variant DM20 [47]. PLP1 and DM20 are solely expressed by oligodendrocytes in the CNS and Schwann cells in the PNS and are crucial components of the myelin sheath [48]. Therefore, a disruption in PLP1/DM20 has detrimental effects on the structure and functioning of myelin. Depending on the type of *PLP1* mutation, histopathology varies, yet some features overlap. There is a significant decrease in the number of mature oligodendrocytes, resulting in a lack of myelin. Altered levels of PLP1 in PMD induce the activation of the unfolded protein response (UPR), which causes apoptosis of oligodendrocytes and neurons [49–51]. The UPR in oligodendrocytes, however, may not be the only neurodegenerative mechanism underlying PMD. Increased astrogliosis and microgliosis have also been observed in brain tissue from patients with different PMD mutations [52]. An astrocytic pathogenetic role is supported by AQP4 redistribution from the perivascular endfeet [45]. Increased levels of AQP4 and its redistribution facilitate oedema formation, as a result of compromised ion-water homeostasis [53, 54]. Neuroinflammation, marked by activated microglia, may also play an important role in the pathophysiology of PMD. The role of inflammation and the NVU, however, has not been further investigated in PMD.

In demyelinating leukodystrophies, the development of myelin is supposedly largely unaffected, yet later in life loss of myelin (demyelination) occurs. Metachromatic leukodystrophy (MLD) is a lysosomal sphingolipid storage disorder inherited in an autosomal recessive manner and caused by genetic mutations in the *ARSA* gene. These result in a deficiency of the enzyme arylsulfatase A (ASA), which is responsible for breaking down sulfatides in the CNS and PNS. Under normal conditions, sulfatides are essential components of myelin, their proper expression being essential for the differentiation of myelinating cells and myelin maintenance. In MLD, accumulation of sulfatides is toxic and results in demyelination. MLD is classified into late-infantile, juvenile, and adult-onset, with disease severity co-varying with age at onset, levels of ASA activity, and type of *ARSA* mutation [55–58]. Microscopy shows demyelination accompanied by accumulation of sulfatides in the glial cells, neurons, and macrophages. There is a negative correlation between increased demyelination and reactive gliosis in the CNS. The involvement of astrocytes has not been investigated extensively, however, one study has demonstrated AQP4 redistribution [45], hinting towards astrocyte dysfunction. Interestingly, in the perivascular space of especially the WM there is accumulation of lipid-laden macrophages, which may release inflammatory mediators that can affect the BBB function [58]. The NVU of MLD patients, however, has not been investigated to such an extent. In other demyelinating neurodegenerative diseases, such as multiple sclerosis (MS), infiltration of leukocytes has detrimental effects on disease progression as the neuroinflammatory cascade worsens and leads to further neurodegeneration together with further BBB breakdown [59].

Another category of leukodystrophies is characterized by myelin vacuolization. In the past years, due to whole-exome and whole-genome sequencing, the number of unclassified leukodystrophies has decreased, resulting in the discovery of a class of leukodystrophies that has deficits in the mitochondrial protein translation [41]. These leukodystrophies are mostly mitochondrial diseases with leukoencephalopathy caused by different mutations in genes related to mitochondrial functioning. Recently, bi-allelic mutations in *LARS2* and *KARS*, which encode for aminoacyl tRNA synthases (aaRSs), were identified as a cause of mitochondrial leukodystrophy [41]. Post-mortem pathological examination of a *LARS2-*related leukodystrophy patient showed loss of myelin, with U-fibres relatively spared. Observation of reactive astrocytes was mostly restricted to the blood vessels, yet reactive gliosis was relatively scarce compared to the degree of WM damage [60]. Pathological investigation of the NVU revealed redistribution of the TJ protein zona occludens 1 (ZO-1) and of AQP4 [45], demonstrating respectively BEC and astrocytic dysfunction.

Astrocytophathies are leukodystrophies caused by mutations in astrocyte-specific genes or in which astrocytes significantly contribute to the disease mechanisms. Alexander disease (AxD) is the prototypic astrocytopathy. It is due to mutations in *GFAP*, which is a cytoskeletal intermediate filament protein that in the CNS is specifically expressed in astrocytes [61–63]. Gain-of-function mutations in *GFAP* cause overexpression of GFAP [64]. Overexpression of GFAP in AxD astrocytes results in the formation of Rosenthal fibres (RFs), which are intracellular protein aggregates that also contain heat shock proteins [65]. RFs cause different cellular dysfunctions, including activation of cellular stress pathways [66], inhibition of proteasome activity [67], and changes in the regulation of autophagy [68, 69]. Not only are the RFs observed in cell bodies, but also in the endfeet and processes of astrocytes, in subpial zones, and around blood vessels where they correlate with BBB dysfunction and contrast enhancement on MRI. A recent study has shown that in AxD, there is astrocytic redistribution of AQP4 and low expression of α-dystroglycan around affected blood vessels [45]. This astrocytic endfeet pathology could mechanically contribute to NVU dysfunction. Besides the formation of RFs, morphology of WM astrocytes is also altered and the astrocytes are enlarged. Also, redistribution of ZO-1 indicates that BECs are dysfunctional. Whether this is a direct consequence of astrocytic dysfunction is still unclear [45]. Neuroinflammation conceivably also plays an important role in the pathology of AxD as perivascular lymphocytic infiltrates have been reported [70–73]. In fact, downstream effectors of the NF-kB inflammatory signalling pathway are significantly upregulated and accompanied by an amount of activated microglia and infiltrated monocyte-derived macrophages in the brains of an AxD mouse model. The same study also confirmed the activation of microglia and macrophages in the brains of AxD patients together with perivascular and intraparenchymal infiltrating T-cells [71]. Yet, the exact role of T-cell infiltration and neuroinflammation in AxD remains an enigma. As mentioned, perivascular accumulation of RFs is a hallmark of AxD pathology, therefore neuroinflammation could be caused by a disruption of the NVU.

Aicardi-Goutières syndrome (AGS) is an autoimmune astrocytopathy characterized by overproduction of interferon alpha (IFN-α), a cytokine activator of the innate immune response. AGS is caused by loss of function mutations in seven genes, including *TREX1*, *RNASEH2A*, *RNASEH2B*, *RNASEH2C*, *SAMHD1*, *ADAR1* and *IFIH1/MDA*, the inheritance pattern of which is mostly autosomal recessive. Mutations in all these genes result in upregulation of IFN-α [74, 75]. Astrocytes are the main cells that produce IFN-α in the CNS and examination of post-mortem brains from AGS patients revealed co-localization of IFN-α and C-X-C motif ligand (CXCL) 10 with GFAP [76]. A study using an in vitro setup with human neural stem cell-derived astrocytes showed that chronic IFN-α exposure reduces cell proliferation while it simultaneously promotes astrocyte reactivity. Correspondingly, genes that are important in the maintenance of WM integrity were altered after exposure to IFN-α [77]. A transgenic mouse model that solely produces IFN-α in astrocytes shows a similar clinical phenotype as AGS patients, including calcium depositions in the basal ganglia, neurodegeneration, seizures, and encephalopathy [78, 79]. Not only does the release of IFN-α in the CNS play an important role in AGS, but also the peripheral inflammation can cross-talk with the CNS. One study indicated an increase of dendritic cells (DCs) in the CSF of AGS patients, more specifically plasmacytoid DCs, which are known to produce large amounts of IFN-α compared to other cell types [80]. Moreover, neuropathology revealed infiltrating T-cells and blood vessel calcifications. More specifically, microangiopathy in the AGS cerebral WM, cerebellum and striatum is accompanied by a thickened tunica media and adventitia in the arterioles and precapillary vessels. Calcification of these cell types can impede vascular constriction and therefore impair CBF, which is crucial for brain functioning [81]. Furthermore, astrocytes have redistributed AQP4 channels, indicating an alteration in ion-water homeostasis, and redistributed ZO-1 proteins, indicating BEC dysfunction [45]. Both in vivo and in vitro data support the notion that astrocytes play a key role in the IFN-α signalling and its downstream pathways and there is strong evidence for a disrupted NVU and BBB in AGS, which could contribute to the pathology.

Megalencephalic leukoencephalopathy with subcortical cysts (MLC) is an infantile-onset leukodystrophy characterized by onset of macrocephaly in early infancy. Recessive mutations in the *MLC1* and *GLIALCAM* genes, respectively encoding MLC1 and GlialCAM/MLC2A, cause MLC [82, 83]. Pathological examination reveals astrocytosis in the molecular layer of the cortex. The amount of myelin within the WM is normal, yet the morphology of the myelin is altered by the presence of countless vacuoles. Also, the myelin sheaths are abnormally thin. Intracytoplasmic vacuoles are also abundantly present in astrocytic endfeet connected to the capillaries. The WM exhibits fibrillary astrogliosis and scarce microglia activation [84–87]. MLC1 expression in the CNS is restricted to the cell membrane of GM and WM astrocytes, ependymal cells, and cerebellar Bergmann glia. In astrocytes, MLC1 is specifically localized in the endfeet at the NVU and glia limitans. MLC1 is important for the regulation of CNS ion-water homeostasis, explaining the increased water content in the patient’s brains [83, 88–90]. A mouse model of MLC revealed that other ion and water channels important at the NVU, including Kir4.1, CLC-2, and AQP4, are also redistributed or altered in expression, indicating that MLC1 regulates the expression of other proteins at the BBB [91]. Not only a defect in *MLC1* but also in *GLIALCAM* can result in MLC. GlialCAM is a chaperone protein for MLC1 and colocalizes with MLC1 in astrocytes. It is also found in oligodendrocytes and axons. As MLC1 proper localization is dependent on GlialCAM, disruption of GlialCAM functioning results in MLC1 dysfunction causing a clinically similar phenotype [82, 92]. Interestingly, a recent study investigated the expression of MLC1 across diseases with a strong neuroinflammatory component: Alzheimer’s disease (AD), MS, and Creutzfeldt-Jacob disease. Here MLC1 upregulation was shown across regions with strong neuroinflammation and increased astrocytosis. Also, the authors demonstrated that MLC1 protein reacts to inflammatory signals, in particular IL-1β, through downregulation of signalling involved in astrocyte activation [93]. Considering that these neuroinflammatory diseases [93] have a disrupted BBB and that MLC1 localization is mainly at the vasculature, it is highly likely that the BBB and the NVU are also disrupted in MLC. Yet, to what extent has to be investigated.

Microgliopathies are leukodystrophies caused by mutations in microglia-specific genes or in which microglia significantly contribute to the disease process. In different neuroinflammatory conditions, microglia secrete inflammatory cytokines that contribute to disease severity. Also, they play a crucial role in (re)modelling synaptic circuits, myelin maintenance, myelin debris clearance, and neuronal reaction upon injury. Adult-onset leukodystrophy with spheroids and pigmented glia (ALSP) is caused by mutations in *CSF1R* encoding colony-stimulating factor (CSF) 1 receptor [94, 95]. Microscopy reveals WM vacuolization and myelin loss. Additionally, there are swollen axons and axonal spheroids present in the WM lesions. These spheroids are immunopositive for amyloid precursor proteins and phosphorylated neurofilaments [96]. Microglia depletion is also seen, together with lipid-laden macrophages and pigmented glia [97–99]. Reactive gliosis is observed in the WM together with AQP4 redistribution, indicating astrocytic involvement in the pathology [45]. CSF1R is a tyrosine kinase receptor crucial for the functioning of microglia. Together with its ligands, CSF1 and IL-34, it regulates production, differentiation, activation, and chemotaxis of microglia and macrophages [100, 101]. IL-34 is an important controller of BBB maintenance and TJ regulation [102], yet whether this pathway is disrupted in ALSP is not known. Oxidative stress also plays a role in ALSP, since high levels of ceroid and iron are found in the macrophages [103–105]. Reactive oxygen species (ROS) indeed induce damage to the BBB in the context of other neurodegenerative disorders and thereby worsen the pathology, yet how this process is regulated in ALSP is unknown.

Leuko-vasculopathies are characterized by involvement of the small blood vessels. These small blood vessels can be at all levels of the CNS vascular tree from the penetrating arteries to the capillary bed. Cerebral small vessel diseases (CSVDs) cause neurofunctional loss and severe cognitive decline usually later in life. CSVDs display a dysfunctional BBB with pericyte degeneration and swelling of the astrocytic endfeet [106–108]. Cerebral autosomal dominant arteriopathy with subcortical infarcts and leukoencephalopathy (CADASIL) is the most common CSVD and is caused by mutations in *NOTCH3*, which encodes a transmembrane receptor that is predominantly expressed by vascular smooth muscle cells (VSMCs) and pericytes [109]. The NOTCH family consists of proteins involved in cell cycle regulation, migration, differentiation, proliferation, and synaptic plasticity throughout the brain [110]. NOTCH3 signalling is in particular important for cell fate specification during embryonic development [111] together with several vascular-related processes in both development and adulthood [112–114]. Mutations of *NOTCH3* in CADASIL change the number of cysteine residues in the extracellular domain of NOTCH3, which then accumulates at the blood vessels. These protein aggregates form, together with other proteins and ECM components, granular osmiophilic material (GOM) that deposits extracellularly in the brain and peripheral organ vasculature, occluding the blood vessels [115]. Indeed, studies have shown a decreased CBF in both patients and animal models accompanied by BBB leakage [116, 117]. Moreover, extensive WM astrocytopathy with AQP4 redistribution is observed together with loss of VSMCsindicating further NVU involvement in CADASIL [115, 118]. The exact underlying mechanism of NOTCH3 dysfunction in CADASIL, however, is still under debate, since some studies using *NOTCH3* knockout mice show that GOMs are not necessary to develop the disease [119–121]. Therefore, more innovative models are needed to study the pathophysiology.

Cathepsin A-related arteriopathy with strokes and leukoencephalopathy (CARASAL) is a recently identified adult-onset leukodystrophy caused by a dominant mutation in the *CTSA* gene encoding for cathepsin A (CathA). CathA is expressed in all tissues throughout the mammalian body but is enriched in the endothelium [122]. CathA is a lysosomal serine protease that degrades intracellular and extracellular substrates and protects β-galactosidase and neuraminidase-1 from intralysosomal proteolysis, thereby stabilizing lysosomal activity. CathA is also involved in ECM formation and stabilization [123, 124]. Axons appear preserved, and loss of myelin, astrogliosis, and preservation of oligodendrocyte density are observed [125]. At the arteriolar branches throughout the cerebral WM and basal nuclei, the vessel walls display asymmetrical fibrous thickening and loss of smooth muscle cells accompanied by occlusion of the lumen. There is a prominent loss of expression of smooth muscle actin (SMA). In some vessels, there is also thickening of the basal lamina [125]. SMA is also a marker for pericytes, pointing towards pericyte dysfunction in this disease. Pericytes might need SMA to contract properly [126–128], thereby regulating the CBF. Changes in the CBF rate could induce dysfunction of the BBB and decrease the quantity of nutrients entering the brain parenchyma [128, 129]. One of the many functions of CathA is the degradation of Endothelin-1 (ET-1), a small signalling peptide regulating vasoconstriction. ET-1 also regulates multiple facets of oligodendrocyte development and response to injury [130, 131]. ET-1 is upregulated in the WM astrocytes of CARASAL patients and results in dysregulation of the developmental programming of oligodendrocyte progenitor cells (OPCs) in the subventricular zone (SVZ) [132]. Further astrocytic involvement has been shown in a recent study that demonstrates the redistribution of AQP4 [45]. This indicates that also ion-water homeostasis in CARASAL could be disrupted. The exact role of CathA dysregulation in the context of NVU functioning, however, remains an enigma.

## In vitro models to study the NVU contribution in physiology and disease

The brain is arguably the most complex organ of the human body, and due to that, there is still much to unveil about the molecular mechanisms and cellular interactions that occur both in homeostasis and disease. Much of what is known about neural development and degeneration has been derived from human post-mortem material and animal models of neurological diseases, such as AD [133], Parkinson’s Disease (PD) [134], and MS [135]. Yet, differences in gene expression, signalling pathways, protein homology, brain architecture, and neuronal circuit complexity between animals and humans reduce the predictive capacity of in vivo models [136–139]. The ratio of WM to GM is also considerably lower in rodents than in humans, a feature that naturally hinders the study of leukodystrophies in these models [140]. These limitations encouraged the development of in vitro strategies that exploit human source material to directly model human physiology.

**Fig. 1:**
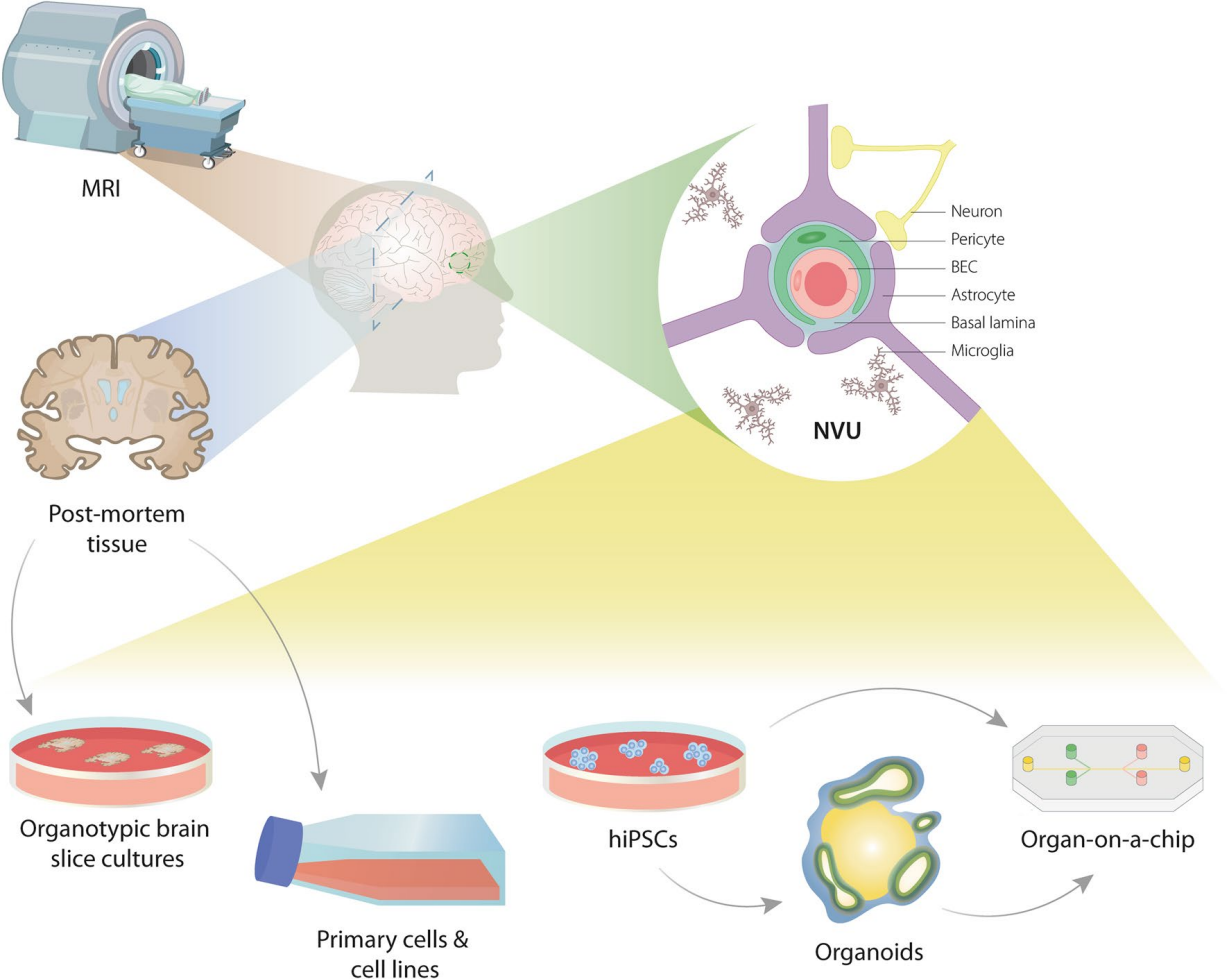
Schematic representation of models to study the NVU in leukodystrophies in humans. The first step of studying leukodystrophies is in a clinical setting, using MRI and next-generation sequencing for initial diagnosis, monitoring disease progression and treatment of patients. Since most leukodystrophies are fatal, post-mortem analysis and post-mortem tissue-derived primary cells, immortalized cell lines, and organotypic slice cultures are valuable tools to distinguish primary cellular processes involved in the pathogenesis. Finally, hiPSC-based models can contribute to investigating the molecular pathways and dynamics at the NVU during disease development and test therapeutic interventions. Together, clinical and basic research can contribute to understanding disease mechanisms in leukodystrophies

Leukodystrophies have been studied for over a 150 years, yet the NVU in these diseases has been overlooked. The first step in the study of leukodystrophies is a proper diagnosis. Nowadays, with the help of MR pattern recognition, new imaging tools, next-generation sequencing, and availability from data worldwide, this process is more rapid and precise. The next important tool in studying leukodystrophies is a pathological examination of human post-mortem tissue. Pathological findings are crucial in determining what underlying mechanisms contribute to disease causation and progression. There are some issues, however, with using human post-mortem tissue. The most important one is that only the end-stage of the disease can be studied. Since leukodystrophies are highly heterogeneous in disease progression and age of onset, crucial disease hallmarks in disease development can be overlooked, especially in the context of NVU dysfunction, which is a process that occurs over time. To overcome these hurdles, proper disease modelling comes into play. In the next sections, we will discuss the current human in vitro models that are used to study the NVU, particularly in the context of leukodystrophies, addressing their advantages and disadvantages and identifying emergent models that better recapitulate the NVU and BBB in physiology and disease. An overview of the in vitro models that will be discussed is displayed in Fig. 1.

### Primary cells and cell lines

Cultures of human primary cells isolated from post-mortem tissues or biopsy samples provided a primary solution for studying the diseases in a human genetic background [141]. However, the limited access, scarce availability, especially in rare disorders as leukodystrophies, and reduced capacity for in vitro expansion, together with phenotypical changes when cultured in vitro, triggered the search for new solutions [142, 143]. Immortalized cell lines, including tumor cell lines [144], overcome the expansion problem but fail to adequately mimic the in vivo cellular behaviour [145]. Even so, all these approaches are still used to investigate BBB physiology and dysfunction [146, 147], angiogenesis in the CNS [148], and the molecular cues exchanged by endothelial and neural cells [149]. The Transwell assay is the most frequently used in vitro assay for replicating the BBB. In this co-culture system, a BEC monolayer is cultured on the apical side of a membrane insert, while supporting cell types, such as pericytes and astrocytes, are cultured on the basal side of the same membrane and also on the bottom of the well. Although this system is ideal for investigating paracrine and autocrine factors secreted by the involved cell types, it fails to model proper heterotypic cell–cell contacts, as well as shear stress and cylindrical geometry, characteristics of blood vessels in vivo [150].

### Organotypic slice cultures

Organotypic slice cultures are an extensively used ex vivo strategy to study the brain, and specifically neurovascular crosstalk [151, 152]. Their main advantage is the ability to preserve structural and synaptic organization [153] in a biologically relevant 3D environment. Numerous applications have been reported, including long-term live imaging, multi-electrophysiological stimulations and recordings, and neurotoxicity assays [153]. It was demonstrated that brain capillaries can survive in organotypic brain slices and, even though they do not present blood flow and are no longer functional, they might still secrete a number or molecules that may influence other cell types in the culture [153]. In an attempt to model the BBB, researchers have cultured brain slices on top of a monolayer of BECs and reported some BBB characteristics in situ [154]. Others tried to understand if the functional and structural properties of the NVU and BBB were preserved in slice cultures [155]. Although immunostainings revealed the maintenance of NVC and vasomotion, BBB intactness can hardly be studied in slices, since the preparation opens up and damages the vessels, eliminating their barrier functionality. Moreover, these slices usually come from animal models, which are not the most reliable replica of human biology [156], while sample scarcity imposes a considerable limitation when studying rare diseases.

### Human induced pluripotent stem cells

The advent of hiPSCs revolutionized biomedical research and became a major tool for studying brain diseases. hiPSCs can differentiate into any cell type and self-renew indefinitely. Due to this, they overcome the expansion and availability limitations of primary cells while maintaining the biologically relevant human context and safeguarding the genetic background of the patient. hiPSCs are induced by over-expressing four genes (Yamanaka’s factors) in easily accessible cells, such as skin fibroblasts [157, 158]. This technology opens up new possibilities to study rare diseases, such as leukodystrophies, for which research is often hampered due to scarcity of biological material.

One of the biggest limitations of hiPSCs is the difficulty to generate fully mature and specific cell types, like excitatory and inhibitory neurons [159] or white and grey matter astrocytes [160]. However, researchers are working hard to tackle this problem and protocols have been developed for the production and maturation of many cell types [161, 162].

Another main limitation of hiPSCs is the resetting of the epigenetic memory. hiPSCs harbour residual epigenetic information specific to their original somatic tissue, which favours their differentiation along lineages associated with the donor cell, while inhibiting alternative cell fates [163]. Nevertheless, several studies have reported the generation of hiPSCs in a more primitive developmental stage, named naïve pluripotency, where the global genome hypomethylation is similar to an early human embryo [164].

In regards to the NVU recapitulation using hiPSCs, it is crucial to acknowledge the heterogeneity of the brain vasculature and surrounding parenchyma, for example in grey and white matter. Regional differences exist in vascular density and function, orientation and permeability, phenotype and function of astrocytes, the ratio of neuronal to non-neuronal cells, and BEC gene expression [165]. Ideally, these distinct aspects must be reflected in the in vitro BBB/NVU model that is used or developed according to the targeted disease, meaning that there is no accurate “one size fits all” BBB model and that the region where the disorder takes place should be taken into account. In the context of leukodystrophies, there is a current need for protocols to generate white matter-specific cell populations, which are already acknowledged as being functionally and phenotypically different from their grey matter counterparts [165].

### Organoids

Using hiPSCs, it is possible to engineer several different strategies to explore brain homeostasis and disorders. A major breakthrough is the technique to culture cerebral organoids [166, 167]. The definition of organoid is still quite controversial, and some researchers struggle with the line between a spheroid and an organoid. Even so, it is generally agreed that, while spheroids are 3D cultures consisting of cell aggregates established from primary cells or immortalized cell lines, organoids are stem cell-derived 3D cell aggregates that self-organize to recapitulate some of the endogenous tissue characteristics [168]. These 3D structures constitute a physiologically relevant tool for drug screening, developmental biology, and pathology, by allowing for cell–cell and cell–matrix interactions in an in vivo-like organization, in contrast to 2D systems [169, 170].

### Organ-on-a-chip

Microfluidic cell culture devices, also known as organs-on-chips (OOCs), have emerged as a system capable of reliably replicating the complex three-dimensional architectures of tissues and organs, including cell–cell interactions and mechanical cues to which the tissues are subject [171]. OOCs are microfluidic systems for cell culture in micrometer-sized perfusable channels, intended to model biological functions and events happening in the different tissues and organs [172]. These chips are usually divided into chambers that are interconnected by microgrooves, allowing for axonal alignment, interactions between different cell types cultured on each of the chambers, or the establishment of molecular spatiotemporal gradients.

OOCs have a substantial number of favourable characteristics as an in vitro model of human physiology or pathologies. One of the most important is the possibility to design culture strategies in which a variety of parameters (cell types and relative positioning; transcellular gradients; cellular alignment; mechanical forces; flow levels and patterns) can be controlled independently while running real-time high-resolution imaging of molecular phenomena in a physiologically relevant microenvironment [172]. The experimental and design flexibility [173] allows precise cell positioning to promote interactions between different cell types while integrating live analytical and microscopical assays.

The ability to perform perfusion cultures with controlled laminar flow in OOCs is especially important for studying the NVU since it can be used to mimic blood flow in microvessels. Besides being directly related to the vasculature, blood flow boosts the function, survival, and differentiation of several NVU-related cell types [174–176]. The inclusion of flow also enables testing of molecular signals and gradients, as well as paracrine/angiocrine cues, which are critical players in BBB maintenance and dysfunction. On the other side, the low number of cells necessary for the culture and the reduced reagent consumption [173] improve the cost efficiency of the system. This feature, allied to the scalability towards parallel automated large-scale platforms [177], provides the basis for the valuable high-throughput characteristic of microfluidics approaches. The smaller relative volume also minimizes dilution of secreted factors that may be critical in the communication between neural and endothelial cells. Moreover, cell phenotypes are more likely to be preserved, since sizes and cell confinement are similar to the in vivo NVU microenvironment [178].

Thus, OOCs represent compelling biological models for studying the underlying pathological mechanisms of several diseases and investigating the function and operation of human tissue structures, including the BBB and the NVU. By combining OOCs with hiPSC technology, it is possible to create a patient-specific tool for precision medicine or to establish a disease model for drug screening or fundamental research. It has already been reported in-chip co-cultures of iPSC-derived BECs with isogenic neural cells (neurons, astrocytes, and neural progenitor cells) [179], astrocytes [180], or pericytes [181], which gradually gets the ultimate goal of building an in vitro human NVU closer to reality.

### 3D bioprinting

3D bioprinting has been appointed as a promising area to produce a complex three-dimensional in vitro NVU in which the contribution of each cell type can be studied at the cellular and molecular levels. Different cell types can be embedded in bioinks—cell-supportive hydrogels with shear-thinning properties—and bioprinted to produce NVU models with various components. Bioprinting enables accurate placing of the vascular and neural cells to establish proper interactive interfaces that recreate the in vivo condition [182].

One of the biggest challenges of 3D printing is the development of bioinks. These hydrogel matrices must have mechanical properties capable of being 3D printed and mimicking the ECM and, at the same time, be suitable for cell viability and growth, while allowing the synthesis and deposition of their own native ECM, to properly replicate the natural microenvironment [183]. For an NVU model specifically, the bioink must enable cellular adhesion and migration, neurogenesis, and vascularization, as well as interactions between vascular and neural components. Besides this complicated bioink formulation, biomimetic 3D bioprinting also poses other challenges, namely the biological complexity of all physical and chemical factors that need to be reproduced and the extensive amount of time needed, since some period of maturation is typically required following tissue printing [184].

### Vascularized organoids and organoid-on-a-chip

Vascularized brain organoids are arising as an interesting approach to assess the cerebral neurovascular crosstalk and mimic the NVU. Different strategies are being developed to produce this kind of organoids. In a pioneer study, whole-brain organoids were generated using patient-derived hiPSCs and then embedded in Matrigel containing isogenic iPSC-derived ECs [185]. In a similar rationale, human umbilical vein endothelial cells (HUVECs) were co-cultured with human embryonic stem cells (hESCs) that were further differentiated into cortical neurons [186]. Using a different approach, researchers induced ETV2 expression on hESCs to guide their differentiation towards the endothelial phenotype and formation of vascular-like structures in human cortical organoids [187]. Recently, a perfusable platform was developed to generate neurovascular organoids from brain organoids surrounded by hiPSC-derived ECs and pericytes [188]. Nevertheless, to date, none of the strategies formed robust lumenized and perfusable capillary-like structures inside the brain organoid nor demonstrated BBB hallmarks such as high TEER, low permeability and expression of BBB markers.

Another strategy that has been recently explored is the integration of organoids in OOC technology. In these organoids-on-a-chip, the advantages of both systems come together in a synergistic model that recapitulates the human body to the fullest. This has been recently done with different organoids, including the brain, for toxicity assessment [189], organoid maturation [190] and vascularization [188]. By constructing a chip with an appropriate design and integrating the right cell types, it is possible, now more than ever, to recapitulate the complexity of the NVU in vitro.

## Future perspectives and challenges in leukodystrophies

There is currently a lack in the availability of advanced human in vitro NVU models for leukodystrophies and other rare diseases, which hinders the development of efficient treatments. Researchers have already reported the generation of region-specific brain organoids—cortex [191], forebrain [192], midbrain [192], hypothalamus [192] and cerebellum [193]—which led to a major improvement on the readout for some region-specific disorders. However, no successful generation of white matter-only organoids was reported so far, which could widely improve the study of and drug development for leukodystrophies. In a different approach, due to their monogenic nature, it would be straightforward to create iPSC lines for the different leukodystrophies by targeting the single disease-causing gene. After this, one should be able to engineer reliable in vitro models of leukodystrophies to further explore the contribution of the NVU and the BBB in these diseases.

By unravelling the cellular and molecular mechanisms behind this intricate crosstalk, new drug candidates may arise, which can then be screened using the same model and tested in a patient-specific manner. A systematic analysis over time can also clarify whether changes in the NVU are a result of the disease, a cause of injury, or an exacerbatory factor of the primary dysfunction, which is not possible to assess in post-mortem studies.

Naturally, optimizing the experimental conditions for such complex models may take time and requires know-how from different fields of research. Therefore, it is crucial to have an interdisciplinary approach to the issue and to establish collaborations with colleagues from distinct backgrounds, to wisely design a robust and reliable model that can effectively replicate human nature.

## Data Availability

Not applicable.

## Abbreviations

aaRSs: Aminoacyl tRNA synthases
ABC: ATP binding cassette
AD: Alzheimer’s disease
AGS: Aicardi-Goutières syndrome
AJ: Adherens junction
ALSP: Adult-onset leukodystrophy with spheroids and pigmented glia
AQP4: Aquaporin-4
ASA: Arylsulfatase A
AxD: Alexander disease
BBB: Blood–brain barrier
BEC: Brain endothelial cell
CADASIL: Cerebral autosomal dominant arteriopathy with subcortical infarcts and leukoencephalopathy
CARASAL: Cathepsin A-related arteriopathy with strokes and leukoencephalopathy
CathA: Cathepsin A
CBF: Cerebral blood flow
CNS: Central nervous system
CSF: Cerebrospinal fluid
CSF1: Colony stimulating factor 1
CSF1R: Colony stimulating factor 1 receptor
CSVD: Cerebral small vessel disease
CXCL: C-X-C motif ligand
EC: Endothelial cell
ECM: Extra-cellular matrix
ET-1: Endothelin-1
GFAP: Glial fibrillary acid protein
GM: Gray matter
GOM: Granular osmiophilic material
hESCs: Human embryonic stem cells
hIPSCs: Human induced pluripotent stem cells
HUVECs: Human umbilical vein endothelial cells
IFN-α: Interferon alpha
IL: Interleukin
IPSCs: Induced pluripotent stem cells
MLC: Megalencephalic leukoencephalopathy with subcortical cysts
MLD: Metachromatic leukodystrophies
MRI: Magnetic resonance imaging
MS: Multiple sclerosis
NVC: Neurovascular coupling
NVU: Neurovascular unit
OAPs: Orthogonal arrays of intramembranous particles
OOCs: Organs-on-chips
OPC: Oligodendrocyte progenitor cell
PLP1: Proteolipid protein 1
PMD: Pelizaeus-Merzbacher disease
PNS: Peripheral nervous system
RFs: Rosenthal fibres
ROS: Reactive oxygen species
SMA: Smooth muscle actin
SVZ: Subventricular zone
TJ: Tight junction
UPR: Unfolded protein response
WM: White matter
ZO-1: Zona occludens 1
